# Taxation of fisheries in Kenya: neither improving management nor raising revenue?

**DOI:** 10.1016/j.wdp.2025.100743

**Published:** 2025-12

**Authors:** Giovanni Occhiali, Olivia Okello

**Affiliations:** a Institute of Development Studies & International Centre for Tax and Development, United Kingdom; b Kenya Revenue Authority, Kenya

**Keywords:** Fisheries, Natural resource taxation, Kenya, Fiscal decentralisation, Sustainable development

## Abstract

Fisheries have long been held to possess significant development potential across Africa, providing food security, livelihoods, and foreign exchange. Debates about their management have centred on the need to close access and on the role of devolution and co-management between central and local government. Because access restriction in practice requires licensing and levies, fisheries’ fiscal treatment lies at the core of their sustainable management. Yet little attention has been given to whether such arrangements in low-income countries achieve either goal. This paper examines the Kenyan case, where fisheries are a devolved sector employing over 1.6 million people. Using a mixed methods approach combining legal and policy analysis, administrative tax data, and 15 qualitative interviews with government officials and stakeholders alongside a focus group discussion, we assess whether Kenya’s fisheries taxation contributes to sustainable management or domestic revenue mobilisation. We find that it does neither. Fragmented regulation, overlapping mandates, and disregard for statutory earmarking prevent levies from funding management. Compliance with general tax obligations such as registration, filing, and payment of income or value added tax is minimal. Reforms should prioritise clearer institutional mandates, stronger coordination across levels of government, enforcement of long-delayed regulations, and targeted action on the sector’s most profitable actors.

## Highlights

• Kenya’s fisheries taxes do not contribute to fund management, raising low revenue.
• Overlapping collection mandate among government levels hinder fisheries governance.
• Only 5.6–5.9 % of registered taxpayers in the sector ever pay any national tax.
• Current data insufficient to calculate fisherfolks total tax burden.
• New regulations in 2024 offer hope, but likely to face implementation challenges.

## 1 Introduction

More than 25 million people are directly and indirectly employed in fisheries and aquaculture across sub-Saharan Africa (FAO, 2020), which contribute between 1.3 % (de Graaf and Garibaldi, 2014) and 2.2 % (World Bank, 2012) of regional Gross Domestic Product (GDP). The sector has long been regarded as developmentally significant: it absorbs rural labour and provides livelihood for those with limited alternatives (Béné et al., 2010, Béné et al., 2016), while generating foreign exchange through exports and fishing agreements (Belhabib et al., 2015, Seto, 2017). Yet, fish stocks in the continent are under heavy pressure, with many already overexploited (FAO, 2020, Zeller et al., 2020). Because fisheries remain open to new entrants, they generate limited resource rents (Cunningham et al., 2009, Willmann and Kelleher, 2009).^1^

Kenya illustrates both the sector’s promise and its constraints. The country’s government has repeatedly identified fisheries and aquaculture as having greater potential for national development (Republic of Kenya, 2007). The sector employs 1.6 million people and contributes an estimated 2 % of foreign exchange (KMFRI, 2018). At the same time, Lake Victoria stocks, providing most of the national catch, are reported as declining; bioeconomic data to underpin management are limited; and illegal, unreported, and unregulated fishing persists (KMFRI, 2018, Republic of Kenya, 2024a). The current estimate of domestic economic contribution – 0.5 % of GDP – likely understates the sector’s value given poor-quality catch data (KMFRI, 2018). Notably, the total annual catch estimate has remained unchanged for a decade (Ministry of Fisheries Development, 2008), despite a probable doubling of participants in the country’s fisheries (Ardjosoediro and Neven, 2008).

A further gap concerns fiscal information: there is no comprehensive account of the taxes and fees collected from fisheries (KMFRI, 2018), a common pattern across Africa (Occhiali, 2023).^2^ This situation undermines fisheries’ valuation and complicates evidence-based policies to support them economically. Most sectoral fees – from vessel licences to fishing permits – are intended to finance management. Only taxes levied under general fiscal legislation contribute to generating revenue (Occhiali, 2023). In Kenya, this includes corporate or personal income taxes from fisheries operators, pay-as-you-earn on formal employees, and value-added tax on processed fisheries product.^3^

However, both management fees and general taxes are often levied on the same, or on closely related, bases. For example, landing fees at beaches, subnational cesses on fish,^4^ and fishers’ income tax assessments by the revenue authority all depends directly or indirectly on catch.^5^ Ideally, assessing both incidence across actors and implications for sustainability therefore requires a holistic view of all charges and taxes at national, county and sub-county levels. Since the 2010 constitutional reform made fisheries a devolved matter, the relevant institutional framework is set by the 2016 *Fisheries Management and Development Act* and the *Fisheries Management and Development Regulations 2024* (Republic of Kenya, 2016, Republic of Kenya, 2024a).

Debates on whether devolution improves fisheries performance and sustainability, including through a more democratic distribution of their revenue (Béné et al., 2003, Béné et al., 2009, Nunan, 2014, Nunan et al., 2015), and on whether a widespread introduction of licences and fees supports their economic valorisation (World Bank, 2004, Cunningham et al., 2009) remain unresolved. Despite this literature, there remains a notable absence of empirical studies on fiscal treatment in low-income countries (LIC) fisheries. This paper contributes to filling this gap by assessing whether the taxation of fisheries in Kenya extracts an appropriate amount of revenue for general use and contributes to their sustainable management. We answer this question through a combination of legal desk review, of in-depth interviews and of analysis of administrative data.

Our analysis indicates that the current fiscal treatment neither mobilises meaningful revenue nor supports sustainable management. To a large extent, this is due to regulatory delay: many implementing measures for the 2016 *Fisheries Management and Development Act* were only gazetted in March 2024, during which time institutions largely continued pre-Act charging practices or left gaps unaddressed.

The regulatory vacuum has also weakened coordination across levels of government and among institutions with stewardship responsibilities. Several bodies lack sufficient resources to execute their mandates; others blur the distinction between revenue that is statutorily earmarked for fisheries management and revenue available for general use. Because fisheries and aquaculture are dispersed, the KRA has historically devoted limited attention to general tax compliance. As a result, the number of registered taxpayers (2,471) remains far below plausible estimates of sector participants (over 110,000), and only a small fraction pay tax. Fiscal support is similarly unorganised: exemptions and subsidies are scattered across instruments, with poorly specified inputs and ad hoc approvals by line ministries rather than automatic qualification.

In this context, existing taxes and fees underperform for both revenue and management. Addressing this requires enforcing the newly gazetted measures and revising institutional arrangements to enable effective inter-level coordination − an endemic challenge in fiscally decentralised settings (Martinez-Vaquez, Lago-Peñas and Sacchi, 2017). As part of coordination, agencies that issue licences and permits should collect information required for KRA compliance processes, while KRA concentrates enforcement on higher-profit actors rather than undifferentiated campaigns.

Evidence on mass registration cautions against broad-based drives: such initiatives are rarely cost-effective and often misfire when aimed at low-income groups with limited access to services (Moore, 2023, Gallien et al., 2023). By contrast, targeted action is more feasible where administrative data already exist − for instance, information on vessels and owners held by the Kenya Maritime Authority.

Finally, if licences are intended primarily as management tools rather than revenue instruments, counties may not be the most suitable issuers. Rural tax collection is frequently ‘messy’ and reliant on informal practices (Prichard and van den Boogaard, 2017), and counties face competing expenditure demands that complicate dedicating licensing revenue to management. Consolidating licensing within a single management-focused institution may therefore better align objectives and accountability.

The remainder of the paper is organised as follows. Section 2 provides a short overview of the tax treatment of fisheries in LICs, with a focus on available Kenya-specific literature. Section 3 briefly describes our methodology, and Section 4 then discusses the current institutional set-up of the fisheries sector, outlining the various roles and responsibilities of national and subnational institutions. 5, 6 present evidence on the use of tax instruments as a tool for fisheries management and to raise revenue, respectively, arguing that they are not currently effective in contributing to either of these. Section 7 presents some potential ways to improve this situation, and Section 8 concludes.

## 2 Literature review

Historically, much of the discussion about fisheries’ tax treatment took place in the context of high-income countries, focusing on their management rather than their contribution to revenue (Gordon, 1954, Scott, 1955, Moloney and Pearse, 1979, Morey, 1980, Clark, 1980). Fisheries management requires sustained monitoring of both ecological and socio-economic variables: fish stocks assessment, identification of spawning areas and of migratory routes, establishment of sustainable harvest levels. In turn, ensuring sustainable harvest involves regularly surveying fishers’ catches, harvesting methods, and fishing areas. All these activities must be carried out in all water bodies where fishing takes place.

Such systems depend on adequately staffed and resourced institutions, which in well-governed contexts may account for up to a quarter of landed value (Arnason, Hannesson and Schrank, 2000). In LICs, however, resource shortfalls are a persistent challenge, with inadequate human and financial resources often leading to widespread illegal, unreported and unregulated fishing (IUFF; Bray, 2000), as well as the persistent vulnerability of small-scale fisheries lacking basic investment in in infrastructure, training and institutional capacity (Dias et al., 2023). Indeed, resource provision is a structural constraint across much of Sub-Saharan Africa (SSA; Occhiali, 2021), with the governance of fisheries in areas as diverse as Ghana (Takyi et al., 2024), Kenya (Kanyange, 2011), and Tanzania (McClanahan, Oddenyo and Kosgei, 2024) all impacted from it.

In high-income countries, the high management cost has led to the proliferation of sectoral subsidies. Given their magnitude, quantified in a substantial body of literature (Sumaila et al., 2019, Sumaila et al., 2016, Sumaila et al., 2010, Arthur et al., 2019, Merayo et al., 2019), it seems in fact highly likely that fisheries in most high-income countries are net fiscal receivers (Occhiali, 2023). Outside a handful of cases in which fisheries have been managed optimally, thereby exhibiting resource rents (Hannesson, 2005, Gunnlaugsson and Saevaldsson, 2016, Gunnlaugsson et al., 2018), there are few economic justifications to levy sector-specific taxes (Gunnlaugsson et al., 2018). Fisheries governance is then guaranteed through a combination of licences and fees expressly levied for its management, combined with general fiscal expenditure to fill potential funding gaps.

In LICs, and SSA specifically, most debates on fisheries taxation are connected to the use of charges and fees as a management tools (Occhiali, 2023). A first strand of the literature focuses on how they can be used to restrict access as a part of a wider sectoral rationalisation needed to reduce overexploitation and ensure that fisheries are appropriately valued (World Bank, 2004, Cunningham et al., 2009, Munro, 2010, Welcomme et al., 2010). Another strand of the literature emphasise fisheries’ capacity to absorb excess rural labour, offering livelihoods to those lacking other opportunities. In this view, closing access would increase poverty more than it would improve economic efficiency (Jul Larsen, 2003, Béné et al., 2010). In practice, fisheries management across SSA blends both these perspectives – following wealth-based approaches on paper, but without completely closing access to fisheries due to welfare considerations (Nunan, 2014).

A second strand examines the consequences of widespread devolution of fisheries management – including revenue raising power – that took place across SSA from the 1980s. This process often entailed dividing fisheries management duties between central government, local governments, and specially created committees representing the whole value chain, called beach management units (BMUs) in Kenya (Béné et al., 2003, Béné et al., 2009, Nunan, 2014, Nunan et al., 2015). BMUs are meant to guarantee some balance in representation of the interests of different actors, with their revenue raising powers supposed to foster democracy and increase sustainability (Béné et al., 2003, Béné et al., 2009, Ribot, 2003). Whether this is achieved in practice is a different matter. While collection practices vary across the continent, BMUs often lack the human resources to collect what they are owed. Increased availability of local revenue does not automatically lead to better quality of management either – accounting practices can be murky, and there are many opportunities for rent-seeking (Nunan, 2014, Etiegni et al., 2017). Indeed, participation in BMUs management is shaped as much by formal state rules as it is by informal local norms, with informal institutions undermining formal mandates at the local level, and formal structures disregarding informal practices at higher levels (Etiegni, Irvine and Kooy, 2020). This disjuncture results in institutional bricolage,^6^ where rules are selectively adapted or ignored, producing governance arrangements that are difficult to hold accountable. This can have severe consequences on both resource access and its sustainability. This is because, despite the existence of formal provision for the inclusion of various types of actors in the management of BMUs, leadership election can be captured by local elites,^7^ leaving ordinary fishers with limited influence and potentially pushing them towards unsustainable practices (Etiegni et al., 2019, Nolan, 2019). While positive examples exist (Cinner and McClanahan, 2015), they seem to be the exception rather than the norm.

A further risk of devolution is that local government will see their management duties as an opportunity for raising revenue rather than as a new responsibility (Béné et al., 2009, Nunan et al., 2015). This seems to be true both when fisheries are kept open access (Béné et al., 2003), and when attempts at economic rationalisation are made (Nunan, 2014). Additionally, this has often led to the emergence of double licensing, where fishers are required to obtain authorisation from multiple authorities for the same activity. This situation can contribute to creating confusion amongst artisanal operators, weakening overall compliance, reducing the coherence of fisheries policy and leading to institutional conflict, as evidenced in the case of Tanzania (Breuil and Grima, 2014, Nakamura and Amador, 2023). Similar issues connected with double licensing have been identified in Uganda, Sierra Leone (Occhiali, 2021), suggesting that this is a frequent consequence of devolved management.

The third relevant strand concerns subsidies towards the sector. Much of this work focuses on estimating the support received by fleets from high-income countries fishing in SSA waters: without subsidies, their operations would not be profitable (Kaczynski and Fluharty, 2002, Witbooi, 2008, Okafor-Yarwood and Belhabib, 2020). LICs subsidise their fleets far less than high-income countries, reinforcing inequitable fishing outcomes (Sumaila et al., 2016, Merayo et al., 2019, Arthur et al., 2019). Most subsidies are deemed environmentally harmful, as they expand fishing capacity with limited regard for the condition of fish stocks (Sumaila et al., 2016, Arthur et al., 2019).^8^ Some, however, can be beneficial, particularly those supporting the establishment of post-processing and value-adding activities (Witbooi, 2008, Occhiali, 2023).

While Kenya-specific literature is limited but instructive. Studies of fisheries co-management on Lake Victoria, including Kenyan shorelines (Nunan, 2014, Nunan et al., 2015, Etiegni et al., 2017), and along the Kenyan coast (Cinner and McClanahan, 2015) underline the difficulty of maximising economic value while retaining fisheries’ welfare-buffer role. Positive examples of co-managed marine reserves exist, yet mandated structures often remain nominal, with frequent institutional bricolage among organisations and risks of elite capture at BMU level.

Recent work on probes institutional relationships underpinning sectoral management. Nunan (2020) shows that the wider political economy around the Lake Victoria shapes fisheries outcome: where decentralised institutions lack resources, fisheries are readily treated as a source of revenue. In Kenya, this situation is exacerbated by an incomplete regulatory framework for both central government agencies and for counties (Ogendi, 2020). Consequently, there is frequent conflict between central and lower-level institutions over the control of fisheries resources and revenue (Mudliar and O’Brien, 2021). Despite double licensing, few services are provided to fishers (Kimani et al., 2020). Tensions between and within BMUs over access and control of fisheries also characterise the Kenyan system with negative implications for resource management (Murunga, Partelow and Breckwoldt, 2021).

## 3 Methodology

Our study relies on three different sets of information to answer this question. First, we reviewed existing policies and regulations for the fisheries sector.^9^ This allowed us to assess the role of different institutions – including who has the right to collect fees and taxes from the sector, and for what – as well as to identify the most appropriate key informants to interview. Second, we gathered published and unpublished data on revenue collected from the fisheries sector from the two county governments whose officials we interviewed. This was complemented by the KRA data on all domestic and customs contributions for taxpayers registered in the fisheries and aquaculture sectors, between 2016 and 2022,^10^ which was first anonymised and then analysed to highlight discrepancies and statistical trends. Third, we conducted 15 in-depths interviews with public and private stakeholders in the fisheries and aquaculture sectors at both the national and county level, and 1 focus group discussion with fishers. Namely, we interviewed two officials from the Kenya Revenue Authority (KRA), one official each from the Kenya Fisheries Services (KFS), the Kenya Maritime Authority (KMA) and the Kenyan Marine and Fisheries Research Institute (KMFRI), one official from the Mombasa County Government and two from the Homa Bay County Government, a senior representative of the Beach Management Units Association, three members of the Alero Beach Management Unit, and a manager from an industrial scale aquaculture farm. All interviews were conducted in English between May and June 2023 by both authors and lasted between one and one and a half hours. Two were conducted online and recorded with the consent of the interviewees, while all others were conducted in person. Two follow-up interviews, which were also recorded, were carried out online in May 2024, following the gazetting of the long-awaited Fisheries Regulations. The focus group discussion (FGD) was held in Mombasa and was attended by one member of the executive committee of the Mombasa Old Town Beach Management Unit (male), one of the youth leaders of the same BMU (male), five fishers from the same BMU (all males), one fish trader (female), and two ‘Mama Karangas’ (fish friers, both female). The FGD was conducted in the presence of both authors in a mix of English and Kiswahili and later translated to English for analysis by one of the authors.^11^

All materials from the interviews and the FGD was transcribed, either from recorded audio or from rich contemporaneous notes, by one of the authors, and then shared with the co-author, who added missing detail and challenged specific inclusions. This negotiated process yielded a consolidated transcript set that formed the basis for analysis. The first author then prepared a thematic condensation, structured around top-level topics that mirrored our interview guide. Analytically, our procedure followed a question-guided thematic synthesis aligned with qualitative content analysis oriented to explanation (Gläser and Laudel, 2013). Rather than line-by-line coding, we selectively extracted material from the consolidated transcript which was relevant to each research questions and organised it into general themes, from which subthemes were developed with specific cases were assigned to each. This process was repeated iteratively, allowing the two authors to confront with each other on what overall framework emerged from the data until an overall agreement was reached (Gale et al., 2013).^12^ Interpretation then proceeded by moving repeatedly between the summarised material and the consolidated transcripts, refining the boundaries of the different themes and sub-themes, and allowing the identification of the pull-out quotes included in the text through immersion-crystallisation cycles (Borkan, 2022). Given the small team size, we did not compute any inter-coder reliability statistics. Rather, we maintain that the credibility of our analysis relied on negotiated transcripts, peer debriefing between authors, triangulation across interviews, FGD, legal-policy texts and administrative records, and the selective use of verbatim quotations in the rest of the paper to ground our central claims.

While we argue that the combination of these different approaches enables us to acquire a thorough understanding of the relationship between fisheries taxation, their management, and their revenue contribution, a few limitations exist. First, it is important to note that one of the authors is employed by the KRA, and that this affiliation was clearly disclosed to all of the respondents. While we stressed with all our respondents that the author was conducting this research for academic purposes and that their anonymity was guaranteed, we cannot exclude that some of their answers may have been influenced by the position of the interviewers. However, if this was the case, it might be more through overemphasising rather than underestimating non-compliance within the sector. All respondents were very also forthcoming in stressing institutional shortcomings with national government’s institutions, including the KRA. Second, accessing data from government institutions other than the KRA has been challenging, either because they were in hard copy (Mombasa County), or because the data could not be made available within the study timeframe (KMA). Third, while we also gathered information on aquaculture from both tax administrative data and interviews, this is mostly analysed through its relationship with the fisheries sector rather than on its own merit.

## 4 The institutional set-up of Kenya fisheries

**Fig. 1:**
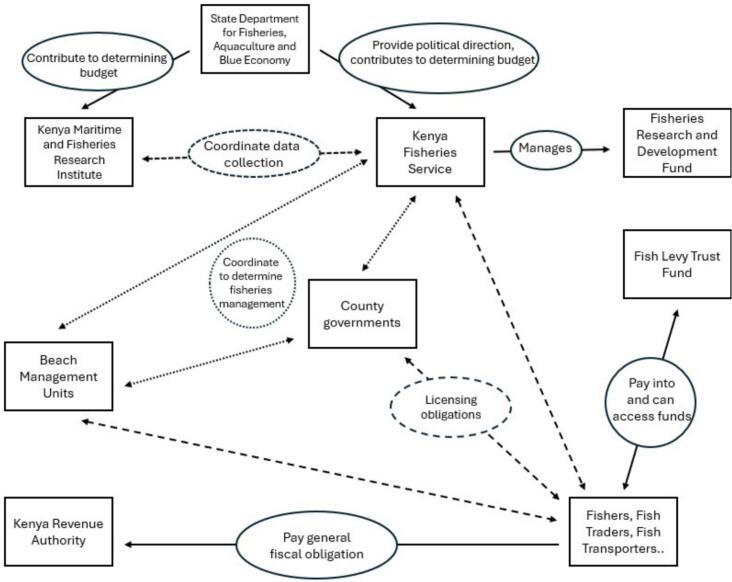
Institutional set-up of Kenya’s fisheries sector Source: Authors elaboration.

The current organisation of the fisheries sector is set out in *The Fisheries Management and Development Act of 2016* (hereafter the Act). The Act establishes a range of bodies under the direction of the State Department for Fisheries, Aquaculture, and the Blue Economy (Fig. 1). Its most significant institutional innovation − superseding the Directorate of Fisheries − is the Kenya Fisheries Services (KFS). Constituted as a state corporation rather than a government department, KFS was intended to enjoy greater operational autonomy. Section 9 assigns KFS varied duties, including promoting fisheries sustainable development (s.9a); formulating policies, standards, and regulations (s.9b-d); collecting and analysing fisheries data (s.9 h); and raising revenue from, inter alia, levies and fees (s.9n).^13^ The Act also establishes two funds − the Fisheries Research Development Fund (s.27) and the Fish Levy Trust Fund (s.28) − respectively financed from royalties payable to KFS and compulsory contributions from anyone engaged in fishing-related activities. The Act further provides for the development of county-specific management measures (s.34–36) and sets the legal basis for regulating BMUs to secure community participation in fisheries’ management (s.37).

Because the 2010 Kenyan Constitution makes fisheries a devolved matter, the Act necessarily provides for the involvement of both counties and BMUs. However, the division of mandates among institutions has been described as ‘create[ing] confusion over the roles and responsibilities of central and county governments’ (Mudliar and O’Brien, 2021: 30). The Constitution tasks county governments with managing fisheries, while assigning the central government responsibility to protect fishing ‘with a view to establishing a durable and sustainable system of development’ (Fourth Schedule, Part 1, 22a. Republic of Kenya, 2010). Where the boundary between these functions lies remains unclear. The Act little to resolve this ambiguity: Section 2 defines ‘fishing’ as ‘searching or taking of fish’, and ‘fishery’ as ‘one or more stocks of fish’ or ‘any fishing for such stocks’, without clarifying institutional responsibilities.^14^

Stakeholders across institutional levels report continued confusion, five years after Mudliar and O’Brien (2021):

“*[Fishing and fisheries] are managed at different levels. However, when you look at it closely again, it is more or less the same thing.*” (Interview with official from KFS)

*“[W]e all have quite similar functions, so there is overlap and it is not clear how to operate when we all have the same role, especially as we don’t always coordinate.”* (Interview with official from Mombasa County)

*“[C]onfusion originated with the devolution process [of the 2010 Kenyan Constitution], as no one knew how to deal with natural resources in that context, but counties wanted a share of the revenue from them, so they got in.”* (Interview with senior representative of BMUs Association)

While Section 7 explores why this confusion persist, it is here worth noting that it has several consequences. Jurisdictional disputes exist between central and local institutions on who should collect which fees – national bodies claiming those linked to ‘fishing’ and counties those related to ‘fisheries’. Similarly, fishers struggle to know which institution to approach for the services for which they get licensed, as the boundaries between support to fishing and to fisheries is blurred, often resulting in suboptimal service delivery.

Overlapping mandates also affect national-level data collection, as both KFS and the Kenya Marine and Fisheries Research Institute (KMFRI) claim authority.^15^ In practice, neither has secure funding to collect reliable data, partly because of this overlapping mandate. Consequently, both rely on unstable donors funding, leading to frequent underestimation of both the catch and number of operating boats, with obvious implications for resource management.^16^

Interestingly, a senior representative of the national BMU association connects the prevailing confusion with the devolution of natural resource management, in turn linked to counties’ desire to access new revenue sources. Much of this confusion originates with an incomplete legal framework and missing regulations.^17^ Both *The Fisheries Management and Development (Marine Fisheries) Regulations 2024* (hereafter *Marine Fisheries Regulations*), and *The Fisheries Management and Development (Fish Levy Trust Fund) Order 2024* (hereafter *Fish Levy Trust Fund Order*), were only gazetted in March 2024 (Republic of Kenya, 2024a). This is despite the former laying out regulations for almost all aspects covered by the Act, and the latter being needed to operationalise the Fish Levy Trust Fund. The legal uncertainty also affected BMUs, which for eight years relied on repealed regulations to guide their activities.^18^ Given the duration of this gap, the confusion − and frequent frustration − expressed by various stakeholders is understandable.

## 5 Fiscal instruments as management tools

The literature indicates that virtually all charges on the fisheries sector outside general fiscal legislation are intended to finance their management. Given Kenya’s current institutional set-up, this would imply that payments to BMUs, county governments and KFS should be earmarked for fisheries sustainable management. In practice, the extent to which this occurs is highly debatable.

Because the *Marine Fisheries Regulations* were only gazetted in 2024, the schedule of KFS licenses – including boat registration, fishers’ licences, import and export permits, and recreation vessels – predated thier inception.^19^ Similarly, no fish levy – potentially as high as 12 % of the value of landed fish for foreign industrial vessels – has ever been collected. All KFS revenue is remitted to the consolidated revenue fund, and KFS operates on budgeted allocations that are typically below the revenue it collects, leading to a reliance on donor funding.^20^ However, even if KFS were allowed to retain its collection, licence revenue is ultimately constrained by biological capacity and would likely be insufficient to cover all mandated activities.^21^ The operationalisation of the Fish Levy Trust Fund might improve this picture, but it is unclear whether it will eliminate the need for donors’ support.

Counties’ capacity to raise revenue from fisheries is tied to their management responsibilities, which remains only partially defined. For example, the Mombasa County government reports providing capacity-building on modern fishing technologies and cites the absence of explicit co-management agreements for the lack of broader support.^22^ This limited engagement is mirrored in the sector revenue performance. Although the county maintains manual, hard-copy records that we could not access directly,^23^ analysis of the county’s *Annual Performance Review Reports* from 2018 to 2021 shows fisheries revenue reported only in 2021, totalling KSh19,788 (US$180.10).^24^ This is surprisingly low given that Mombasa has 14 BMUs managing 50 landing sites (Menza and Mange 2020), and that the Mombasa County Bills 2020 lists 28 separate charges for the sector (Appendix A, Table A1.1).

By contrast, Homa Bay County plans to provide weighing units (60 of which have already been installed), toilet facilities, and potentially electricity to the BMUs on its territory, though providing some of these might fall outside its mandate.^25^ This greater engagement is reflected in sectoral collection: Table.1 shows fisheries contributing, on average, 4.3 % of the county’s own revenue between 2018 and 2023.

**Table 1:** Fisheries revenue collected, Homa Bay county, 2018–2023.

Fiscal year	Fisheries revenue	% of Total collection
2018/2019	4,071,105	4.55 %
2019/2020	5,356,153	4.52 %
2020/2021	5,187,524	4.27 %
2021/2022	6,830,580	4.66 %
2022/2023 (Q1-Q3)	5,476,766	3.43 %

Source: Authors elaboration on unpublished data from Homa Bay County.

As we can see from these two cases, revenue from fisheries varies from being virtually irrelevant in Mombasa, to being one of the five most important sources of revenue in Homa Bay. How contributions from the sector are conceived also seems to vary. In Mombasa, it appears to be squarely considered as revenue, despite its currently limited significance:

*“There are expectations that the fisheries department will contribute to revenue mobilisation for the county, but we do not have an official target per se. They ask us how much we think we could mobilise and we tell them, so we are the one setting it.”* (Interview with official from Mombasa County government)

On the other hand, in Homa Bay there was an appreciation of the connection between collection from the sector and its expenditure:

*“We collected… as of April, KSh5.8 million in 2022*–*23 – these are from fish cess only, as the other charges go into trade revenue or business permit revenue. Even adding those, I doubt we ever got to KSh10 million. It is easy to see that these are not sufficient if one compares the requested expenditure on the sector.”* (Interview with officials from Homa Bay County government)

While there is some justification for the view that counties treat fisheries chiefly as a revenue source,^26^ the picture is more complex. Where the sector is economically significant, as in Homa Bay, revenue extraction is understood to require some service provision. Several charges in the Homa Bay County Bills 2020 are reportedly enforced ‘very gradually to maintain social peace’,^27^ demonstrating an ad hoc and cooperative process to compliance. This is not unusual: subnational revenue collection outside metropolitan areas is often ‘grounded in existing social relationships and collective norms’ (Prichard and van den Boogaard 2017: 172), which can strengthen state-society relations. Something similar seems to be happening: legislated fees may be unofficially waived when service provision is recognised as suboptimal.

The above quotes also distinguishes across charge types. The fish cess, levied directly on fishers at landing, is viewed as a substantially different from market-use fees or trading permit, and they are indeed listed separately in the Homa Bay County Bills (Appendix A, Table A1.2). While understandable from an accounting perspective, such separation complicates assessment of cumulative charges^28^ along the value chain and their effect on the price that fishers can obtain for the primary product.^29^ A comparison of the fisheries schedules of Homa Bay and Mombasa County Bills shows substantial differences in both the level and type of charges. Because their stewardship mandate does not differ, this reinforces the inference that management responsibilities are not a primary consideration when charges are set.

Finally, BMUs also have a mandate to raise revenue from their members, who range from fishers to net-menders and fish traders. Each BMU adopts by-laws specifying charges, usually including registration fees for its members, boat licensing, and fees for landing and weighing of fish^30^ – two examples are presented in Appendix A, Table A1.3 (Alero BMU) and Table A1.4 (Kilifi BMU). Although fee schedules vary, BMUs’ main source of revenue has historically been fish landed on their shores, determining the resources available for management activities or members welfare.^31^ However, some counties already levy additional fees on landed catch, and KFS will soon do the same following the promulgation of the *The Fish Levy Trust Fund Order 2024*. BMUs are therefore considering whether they could increase their reliance on revenue from traders.^32^ Regardless of source, how much revenue is collected is known only to BMU members and subject to a ‘social audit’, whereby members scrutiny the accounts, with the results not shared with other bodies, including their national association.^33^ While this might appear to be less than ideal, members value BMUs’ mediating role with the many organisations from licences are required.^34^ BMUs importance is widely recognised,^35^ and they are seen as essential for fisheries management,^36^ having demonstrated significant convening power. In turn, this grants them bargaining power regarding the services expected by their members in exchange for the mandated levies.^37^

In summary, fiscal instrument do not presently appear to contribute to better fisheries management. Nearly a decade after the Act was approved, neither fund it establishes is operational. The lack of up-to-date regulations has also deprived KFS of earmarked resources, fostering dependence on donor support. Counties, constitutionally mandated to steward fisheries, have introduced their own charges, but these are not clearly tied to management strategies, in part because precise responsibilities remains unclear. Some charges, such as market fees for fish traders, are general-purpose subnational revenue. Others – such as licence fees for various operators – should be earmarked for fisheries management, and it is unclear whether this occurs. BMUs are the one actor explicitly raising revenue for management, yet their main revenue base is being encroached upon by counties and KFS. While discouraging, even the best managed fisheries can be net revenue receivers. The sector’s contribution to the general fiscal purse, examined next, can support the case for greater state investment.

## 6 Fiscal instruments as (national) revenue-raising measures

Given how such instrument are conceived, it may be tempting to treat all charges and fees paid by fishers and related actors to governments entities as part of their revenue contribution. Yet, many of these charges and fees are, at least in principle, management tools: they should not flow to the consolidated revenue fund but bye used exclusively to ensure fisheries’ sustainable management. Hence, while it might represent a small – perhaps the smallest – share of what fishers pay to public bodies, the tax contribution of fisheries to the KRA under general legislation should be assessed on its own merit. Because many sectoral bodies lack public funding, a clearer picture of tax revenue collected from fisheries could strengthen the case for greater support to the sector.

As of 2022, 2,741 taxpayers were registered in the fisheries and aquaculture sectors – 61.5 % in fisheries, and 38.5 % in aquaculture. The vast majority (78.7 %) are incorporated; individuals are more prevalent in aquaculture (32.4 %) than in fisheries (14.3 %). The prevalence of incorporated entities helps explain why most taxpayers are managed by the Nairobi tax office (18.6 %) rather than Kisumu tax office (11.7 %) – Kisumu is the largest Kenyan city near Lake Victoria. Registration takes place where management is located, rather than the activities. Comparing registered taxpayers in the fisheries sector – 1,685 – with the number of fishers estimated to be active by KMFRI (14,000; KMFRI 2018), or by KFS (65,000; KFS 2023), suggests widespread non-compliance. A similar gap exists in aquaculture sector, which has 1,056 registered taxpayers versus an estimated 70,000 aquaculture farmers (KFS 2023).

Two complementary explanations help account for the discrepancy between active and registered actors. First, sectoral information capture in the KRA’s iTax (domestic IT) system is often poor.^38^ This is because sectoral information is viewed as only relevant for the Kenyan National Bureau of Statistics. Therefore, the KRA cares little about the reliability of information about industrial division and group – respectively the 3- and 4-digit international standard industrial classification (ISIC) – provided by taxpayers at registration.^39^ In our data, between 58.6 % and 61.8 % of fisheries and aquaculture taxpayers display a mismatch between industrial ‘division’ and ‘group’ depending on the extraction criterion. Hence, it is likely that some taxpayers industrial category was misclassified at registration, leading to their exclusion from the analysis.

Second, compliance with registration obligations among fishers is reportedly very low.^40^ For many, fishing is conceived as a traditional occupation. Fishers ‘were raised as fishermen; they do not approach the profession with a commercial spirit’.^41^ Compliance with registration and licensing requirements is low, and resisted.^42^ Neither counties nor BMUs ask for Personal Identification Numbers (PINs) at registration, fearing further dampening of already low compliance levels^43^: a senior member of Alero BMU estimates that only about 20 % of their members are likely to have a PIN.^44^ As counties and BMUs are the institutions with the richest sectoral information, their decision not to capture PINs during licensing reduces the usefulness of their data for compliance purposes. A similar disregard for compliance-relevant information can be seen at the national-level: none of the forms included in the 2024 *Fisheries Management and Development Regulation* ask for PINs. The only exception to this is KMA, which since 2018 has required boat owners’ PIN during compulsory safety assessments of seafaring vessels.^45^ Given this landscape, the small number of fishers in the KRA register is unsurprising.

Overall, compliance with many tax obligations is low. As Table.2 shows, very few taxpayers in the sector paid tax in any given year. Discrepancy between the two panels of Table.2 again reflects the poor quality of industrial classification data in KRA’s iTax system, which complicates determining both how many taxpayers ever paid and total sectoral collection. Given that at most 227 unique PINs made a payment between 2016 and 2022 – 8.28 % of those registered – compliance is clearly broadly weak. Unsurprisingly, compliance rates vary across taxpayer categories. Those managed by the Large Taxpayers Office pay regularly, 71.4 % of those managed by the Medium Taxpayers Office paid at least once, and compliance rates drop significantly after that.^46^ By contrast, none of the 583 individual taxpayers in fisheries and aquaculture made a single tax payment over the period. Administrative data thus confirms KRA stakeholders’ view that sectoral challenges concentrate amongst small single-owned companies, whose owners equate ‘tax payment as their own losses’, and individual fishers, who keep limited book and transact at landing sites.^47^ KRA officials also note that many individual fishers likely fall below the tax threshold. Enforcement efforts should therefore prioritise actors selling fish in bulk to restaurants or processing companies.^48^ Boat owners should also be targeted, as they are probably the most profitable actors in the sector.^49^

**Table 2:** Number of taxpayers making at least one tax payment each year and total revenue collected from domestic taxes, ISIC 3 and ISIC 4 data extraction.

Year	Industrial division (ISIC 3)		Industrial group (ISIC 4)	
	Unique PIN	Total revenue	Unique PIN	Total revenue
2016	35	141,265,674	58	231,352,314
2017	37	148,930,586	57	334,225,891
2018	46	218,096,856	72	359,948,878
2019	40	246,109,531	88	298,737,718
2020	51	178,551,398	99	266,836,246
2021	53	197,127,136	111	336,732,430
2022	50	239,922,799	138	434,327,313

Source: Authors analysis of KRA data from the iTax system.

The potential scale of tax expenditures on the sector also warrants attention. The exact amount of revenue foregone to support the development of these sectors is currently unknown. Although Kenya recently started publishing tax expenditure reports, the information contained is too aggregated to be useful. Direct computation is difficult because of how exemptions are coded in law. For instance, the East African Community Customs Management Act exempts *‘[i]mported inputs and implements by persons engaged in horticulture, aquaculture, agriculture or floriculture* ***as recommended by the responsible Minister in a Partner State and subject to conditions***’ (emphasis added). Domestic provisions are similar, with the Finance Act No. 8 of 2021, s. 27(a)(xxv), granting VAT exemptions on taxable supplies including ‘*fish feeding and handling, water operations, cold storage, fish cages, pond construction and maintenance, and fish processing and handling, imported or purchased for direct and exclusive use* ***on the recommendation of the relevant state department****’* (emphasis added).

Such wording gives line ministries substantial discretion over eligibility and scope. Apart from reducing the transparency and accessibility of these fiscal incentives, better-connected firms are more likely to have a significant share of their inputs exempted.^50^ This discretion also impedes measurement: without standardised reporting, quantifying tax expenditure requires cataloguing all ad hoc requests granted by ministries and state departments or sifting through individual import transactions from the customs system.

Even so, our attempt at quantification suggests that expenditure could be substantial. Table 3 indicates that, between 2017 and 2021, 58.4 % of import duties and import VAT owed by six selected taxpayers was exempted, amounting to an expenditure of KSh76.6 million. Over the course of 2022–2023, 27.4 % of import duties and import VAT due by six selected taxpayers (three overlapping with the previous period) were exempted, for a total of KSh54.2 million. These figures cover only import duties and import VAT, and do not capture tax expenditure on domestic taxes, which are even harder to assess.

**Table 3:** Import duty and import VAT exemptions 2017–2023, selected taxpayers.

	2017–2021			2022–2023		
	Tax remitted	Tax computed	Exemptions	Tax remitted	Tax computed	Exemptions
Taxpayer 1	370,304	589,053	37.14 %	−	−	−
Taxpayer 2	12,289,464	28,466,688	56.83 %	6,483,042	11,074,586	41.46 %
Taxpayer 3	181,110	772,027	76.54 %	−	−	
Taxpayer 4	41,503	1,137,351	96.35 %	−	−	
Taxpayer 5	41,141,338	99,189,847	58.52 %	126,681,112	170,179,479	25.56 %
Taxpayer 6	649,356	1,155,854	43.82 %	5,126,010	8,515,110	39.80 %
Taxpayer 7	−	−	−	826,830	2,798,782	70.46 %
Taxpayer 8	−	−	−	4,163,304	4,852,168	14.20 %
Taxpayer 9	−	−	−	703,318	798,100	11.88 %
Total	54,673,075	131,310,820	58.36 %	143,983,616	198,218,225	27.36 %

Source: Authors elaboration on data from Simba and ICMS systems.

## 7 Ways to increase compliance, raise more revenue, and manage fisheries better

The preceding discussion shows that fiscal instruments applied to fisheries are sub-optimally for both management and raising revenue. Causes range from counties treating licences as revenue-raising instruments to weak KRA data management and enforcement practices. Nonetheless, avenues for improvement exist. Unsurprisingly, most involve strengthening the sector institutional set-up, as also noted by Mudliar and O’Brien (2021). Many obstacles were also acknowledged by the Kenyan Government’ regulatory impact assessment published as our study concluded (Republic of Kenya, 2024b), signalling scope for reform.

First, mandates of different government agencies require clarification and streamlining. Confusion persist about specific duties, which must be directly addressed.^51^ In particular, the distinction between ‘fisheries’ and ‘fishing’ needs to be clarified, starting from the Act. This will allow county governments to know their precise responsibilities, and fishers to know who should they approach for particular services. A structured coordination forum for all institutions involved is also required. The most recent sector-wide meeting, convened by BMUs to question the legal basis for county levies, was an ad hoc effort.^52^ One regulations gazetted in March 2024 seeks to ‘facilitate proper consultation between the County… and the National Government on matters that promote fisheries management’ (Republic of Kenya, 2024c: 39). Because coordination in devolved systems is often difficult (Martinez-Vasquez et al., 2017), it remains uncertain whether this provision will materially improve coordination.

Second, harmonising data collection efforts should be a priority. A holistic and consolidated view of the sector’s activities supports both management and compliance. This is true for both fiscal-related and catch-related data. Currently, catch data are collected by BMUs and KFS, while vessel counts involve KMA, KMFRI, and KFS. However, both the KMFRI and KFS lack funding for systematic surveys of fisheries’ stocks and economic actors which should underpin the development of management strategies for each water body.^53^ Similarly, BMUs recognise the value of the data they collect on catches, but have little incentive – and even less institutional support – to improve their practices.^54^ Better catch- and boat-level data would inform county-level estimation of revenue from licences and fish cesses, in turn strengthening counties’ incentives request PINs at licensing. The recently gazetted regulations explicitly task BMUs with collecting data on catches from their members. However, this responsibility emerges without commensurate own-revenue capacity (Republic of Kenya 2024b, c). It remains unclear whether implementation will yield substantive change.

Third, once data improve, cumulative fiscal burden faced by different fisheries actors should be assessed. This should happen alongside the estimation of how much is ostensibly collected purely for management purposes. Many fishers reports being taxed by an excessive number of institutions,^55^ with some respondents noting that this excessive fiscal pressure may reduce fishers’ net income. By itself, this might also encourage overfishing,^56^ although boat owners’ profit motives are probably more influential.^57^ For most fishers interviewed, it was evident that all levies and charges count as “taxes”, regardless of who is levying them or for what reasons. It seems highly likely that current low tax compliance could partially be explained by their perception of already contributing enough. This is especially relevant now that The *Marine Fisheries Regulations 2024* and *The Fish Levy Trust Fund Order, 2024* have come into force, layering new charges onto existing ones.

Fourth, increased investment in basic amenities, service delivery, financial support to develop post-harvest activities, and training and capacity-building would both increase revenue potential and facilitate management.^58^ The government recognises these challenges and cites them among the reasons for promulgating the long-waited regulations of 2024 (Republic of Kenya, 2024b). Clarifying management responsibilities of various institutions and mapping how much is levied from whom are prerequisites for determining whether current investment matches sectoral contributions. Even where it does, many fisheries worldwide are net fiscal receivers, so additional funding from general revenue might still be warranted. Given concerns about purely targeted exemptions,^59^ some funding might already be available for reallocation towards more efficient uses.

Finally, investment should precede, or at least accompany, any push on sectoral enforcement. Several fishers expressed strongly negative views of KRA,^60^ and maintained that the national government is doing little to support their activities and livelihood. Because many of them are likely below the taxable threshold,^61^ KRA should prioritise sensitisation and encourage voluntary compliance. Evidence on mass tax registration campaigns show they rarely deliver promised benefits (Moore, 2023, Gallien et al., 2023). While these studies do not focus on sector-specific registration drives, their results likely apply. While the temptation of indiscriminately registering all artisanal fishers for tax purposes should be resisted, there is an argument for more targeted interventions.

Many of the problems associated with the use of the term ‘informal sector’ (Gallien, 2024) mirror those of the term ‘artisanal fisheries’, which contain very heterogenous actors (Smith and Basurto, 2019, March and Failler, 2022). In Kenya, boat owners and fish traders are frequently identified as those earning most profit in artisanal fisheries, accruing very significant income from their ‘small-scale’ activities.^62^ Actions to increase their tax compliance seem well-justified. Many boat owners can likely be identified through KMA data, which includes their PINs.^63^ Additionally, both county governments and BMUs should have information on fish traders, albeit without PINs. Institutional structures for data sharing are not currently in place, and data exchange relies on goodwill, upon request.

That such a seemingly dysfunctional set-up of the fisheries sector persisted for almost a decade suggests the existence of significant reform constraints. Indeed, some interviewees suggested that this system enabled both political and financial extraction. Legal ambiguity allowed county politicians to promise their constituents unlimited fish harvests, giving them reasons to oppose any change.^64^ Concurrently, counties could create parallel licensing structures, often at higher rates than central charges, securing additional revenue.^65^ Bureaucratic complexity also slowed reform. The Kenyan Constitution requires extensive stakeholder consultation for almost any proposed change in legislation. The Act was held up in court in 2023 over alleged insufficient consultation on banned artisanal methods, reportedly backed by a coastal politician.^66^ It was through this consultation that the regulations stalled. It gave counties a platform to stall any attempted reform threatening both particular interests and a source of own-revenue.^67^ The concentration of fishing in areas perceived as hostile to the ruling party further reduced incentives to complete regulation.^68^

Even so, the new regulations were eventually approved in early 2024. After a long delay, political interests appear sufficiently aligned to allow action in the sector.^69^ Much of the impetus seems to have originated from cabinet, suggesting increased political salience.^70^ A window now exists to address remaining shortcomings, many of which are acknowledged by the government (Republic of Kenya 2024b). While promulgating the new regulations is a significant first step, they do not address all the problems identified in this study. It is clear that further work is required to strengthen sustainability in the sector.

## 8 Conclusion

Existing research on fisheries in LICs has largely examined their welfare buffer role (Jul Larsen, 2003, Béné et al., 2010),), the potential for closing access through licence issuance to reduce overexploitation and foster economic rationalisation (Cunningham et al., 2009, Munro, 2010, Welcomme et al., 2010), and on the impact of management devolution (Béné et al., 2003, Béné et al., 2009, Nunan, 2014, Nunan et al., 2015). While this literature touches on fiscal treatment – licensing raises revenue and devolution shifts who collects it – empirical assessments of whether fiscal arrangements improve management, including by mobilising sufficient revenue, remain scarce.

We address this gap by focusing on Kenya, where fisheries employ over a million people, contribute to foreign exchange earnings and are identified as a sector with high growth potential (Republic of Kenya, 2007, KMFRI, 2018). Despite various management reforms, including devolution in 2010 and the creation of a new institutions in 2016, the sector economic contribution appears stagnant. Part of this may reflect poor-quality data (KMFRI, 2018): catches are likely underestimated and neither the total number of boats nor that of active actors is known. This lack of data makes developing management strategies extremely challenging. Because many charges and fees are levied on catch or arise from vessel and fisher licensing, weak data also impede assessment of whether fiscal measures support sustainability, including by raising adequate resources for management.

Our analysis suggests that these goals are not being met. Devolution and the *Fisheries Management and Development Act of 2016* expanded management duties to county governments and KFS but did little to clarify their respective roles. KFS, the main national body overseeing fisheries’ organisations and development, struggles to secure sufficient funds to deliver on its overarching mandate. Partially, this is because the regulations enabling its revenue raising capacity were only gazetted 8 years after its creation. Counties have introduced numerous charges and fees across fisheries value chain yet often treat them as a general revenue source rather than as a management tool. With stewardship responsibilities unclear, reinvestment appears driven more by local economic salience than by explicit management plans. BMUs, operating for years under repealed regulations now replaced, earmark their collections for management but increasingly face encroachment on their revenue base.

KRA, for its part, has not prioritised compliance from a geographically dispersed sector in which many actors likely fall below the income tax threshold. Analysis of tax administrative data – despite ISIC coding issues that warrant separate remedy – show that only a small share of active fisheries and aquaculture actors register for tax, and an even smaller share of registrants pay, indicating very low compliance. It is similarly difficult to judge the development impact and revenue cost of sectoral tax expenditure as eligibility rests on poorly defined inputs and high ministerial discretion rather than automatic qualification.

There is therefore ample scope to improve the effectiveness of the fisheries’ fiscal treatment for both revenue and management. Overlapping mandates should be minimised through clearer duties; the recently gazetted regulations are a useful starting point but do not obviate the need for further refinement. Establishing a coordination forum for institutions with stewardship responsibilities can facilitate information sharing needed for both proper management planning and to obtain a holistic view of collection. This would help to understand the total fiscal burden faced by sectoral actors, which can inform a compliance strategy for the KRA. The latter should start with extensive sensitisation and targeted enforcement on boat owners and fish traders (identified as those with higher profits), rather than mass registration of fishers. Absent such steps, fisheries are unlikely to contribute to national development in the way long envisaged by the Kenyan government.

## CRediT authorship contribution statement

**Giovanni Occhiali:** Writing – original draft, Validation, Project administration, Methodology, Investigation, Funding acquisition, Formal analysis, Data curation, Conceptualization. **Olivia Okello:** Writing – original draft, Validation, Investigation, Formal analysis, Data curation, Conceptualization.

## Funding

This research was supported by funding from a 10.13039/100000865Bill & Melinda Gates Foundation Grant (OPP1197757), a 10.13039/100007843Norwegian Agency for Development Cooperation Grant (QZA-17/0153) and a UK Aid Grant (300211-101).

## Declaration of competing interest

The authors declare that they have no known competing financial interests or personal relationships that could have appeared to influence the work reported in this paper.

## Figures and Tables

**Table A1:** Fisheries charges, Mombasa County Bills 2020.

No.	Particulars	Ksh (2020/21)
	**101. REGISTRATION OF LOCAL FISHING VESSELS**	
1022	A-Non-mechanized fishing vessels	200
1023	B-Mechanized fishing vessel	200

	**102. FISHERMEN AND SPORT FISHING LICENCES**	
	**Fisherman’s licence**	
1024	Class A − Fisherman not using any craft	200
	Class B − Fisherman using non-mechanized vessel:	
1025	Vessel not exceeding 5 (five) metres length	200
1026	Vessel exceeding 5 (five) metres length	200

	**Class C**	
	**Fisherman using mechanized vessel:**	
1027	Vessel of more than 5(five) metres length and 1½(one and half) metres beam.	200

	**Sport Fishing License**	
1028	Annual License	3000
1029	Monthly License	1000
1030	Fortnightly License	500

	**Miscellaneous Licences/Certificates:**	
1031	Aquarium fish dealer’s licence per annum	
	Crustacea dealers licence: per annum	
1032	Wet	2000
1033	Dry and live crabs	1000
	Fish movement permit for:	
	(1) Vehicle	
1034	(a) Not exceeding 3 (three tones) per trip	500
1035	(b)Exceeding 3 (three tones)per trip	1000
	(2) Boats	
1036	(a) Not exceeding 10 per trip	1000
1037	(b) Exceeding 10 per trip	5000
1038	(3) Luggage accompanying trader using public passenger carriervehicle per trip	100
1030	Fish processing licence per annum − Fish	2000

	**Shops**	
1040	Fish processing licence per annum − Small and medium Fish	5000

	**Factories**	
1041	Fish processing licence per annum − Large fish factories	10,000
1042	Fish export fee at 0.5 % (Zero point five percent)	N/A

	**Ad valorem of market price per trip**	
1043	7 tonnes and above lorry	200
1043	Fish Trader Licence per Annum	200
1044	Beche-De-Mer Trading Licence Per Annum	100
1045	Shell Dealers Licence	3000
1046	Shell Collectors Licence	200
1047	Registration of a Fishing Club/Association	5000
1048	Boat Operation Charges	15 per kg of fish

**Source:** Kenya Gazette Supplement No. 10 (Mombasa County Bills No. 6), MOMBASA COUNTY BILLS, 2020, NAIROBI, 30th November 2020.

**Table A2:** Fisheries charges, Homa Bay County Bills 2020.

Market user charges	2020 /202I charges	
Fish (omena) one trough (approx 10 kg) and below	10	
Fish omena (half a sack) (approx 20 kg)	30	
Fish omena full basket (approx 50 kg)	60	
Fish Omena extra lull size (approx 90 kg)	100	
Nile perch one trough (approx 10 kg)	20	
Nile perch hall basket (approx 20 kg)	50	
Nile perch tull basket (approx 50 kg)	80	
Nile perch extra full basket (approx 70 kg)	200	
Tilapia one trough (approx 10 kg)	40	
Tilapia half a basket (approx 25 kg)	80	
Tilapia full basket (approx 30 kg)	100	
Tilapia full basket (approx 50 kg)	150	
Tilapia full basket (leaving Homa bay county) (approx 50 kg)	200	
Nile perch per ton (1,000 Kg)	2000	
Other fish species one trough (approx 10 kg)	30	
Other fish species half basket (approx 20 kg)	100	
Otherfish species full basket (approx 50 kg)	150	

**Fisheries Charges**		
**Sale of aquatic fish**		
Sale of aquatic fish 6–10 cm long	Per fish	20
Sale of aquatic fish 11–15 cm long	Per fish	20
Sale of aquatic fish 16 cm and above	Per fish	30
**Annual License**		
Fish processing	Annually	3000
Fishing camp	Annually	20,000
Sport fishing	Up to two weeks	200
Sport fishing	Per month	500
Sport fishing	Per year	1000

Source: Kenya Gazette Supplement No. 14 (Homa Bay County Bills No. 5), HOMA BAY COUNTY BILLS, 2020, NAIROBI, 28th September 2020.

**Table A3:** Alero BMU fees.

S/No	Activity	Fees in Kshs	Frequency
1	Application Fees	500	Once
2	Membership Fees	100	Once
3	Annual Registration/Membership	130	Annually
4	Vessel Registration	150	Annually
5	Landing Fees • Members • Non Members	• 2 • 4	Per kg
6	Trucks Loading Fees • Members • Non Members	• 300 • 500	Per Day
7	Gear Counting Fee	50	Per vessel per Quarter
8	Plot allocation	500	Per 10 foot
9	Toile Maintenance	10	Weekly

Source: Authors’ translation from Alero BMUs by-laws, unpublished.

**Table A4:** Kilifi BMU fees.

S/No		Activity			Fees in Kshs		Frequency
1		Membership Fees			200		Once
2		Persons undertaking any business at the beach(Groups)	Registration		1000		Once
			Renewal		500		Annually
3		Persons undertaking any business at the beach(Individuals)	Registration		200		Once
			Renewal		200		Annually
4		Placing a container for any trade at the beach e.g. hotels, fish trucks, trucks for goods (foodstuffs, stones etc.)			100,000		Monthly
5		Restaurants (tents), open markets			30,000		Annually
6		Setting up lobster traps			6,000		Annually
7		Vessel docking for loading or unloading cargo/fish(non-members)			6,000		Per trip
8		Boat Repairers a. Local member – 1500 b. Non-members (1–5 tonnes capacity) – 5,000 c. Non Members, 5 tonnes and above – 10,000			a. 1500 b. 5,000 c. 10,000		Once
9		Freezer/cooling plant charges			a. Kshs. 5 per kg for 24 h b. Kshs. 10 per kg for Non Members for 24hrs		
10		any random traders at the beach			10		Daily
11	Docking			1000		Per trip	
12	Fish landing − Members			3		Per kg	
	Fish landing − Non Members			5		Per kg	
13	Lobster landing – Kshs. 10 per kg			10		Per kg	
14	Crabs landing – Kshs. 5 per kg			5		Per kg	
15	Vehicles parked at the beach – Kshs.250			250		daily	

Source: Authors’ translation from Kilifi BMUs by-laws, unpublished.

**Table B1:** Codebook.

Theme	Sub-theme	Definition	Include when…	Exclude when…
Sectoral organisation	Management, county level	County governments’ role in post-landing activities; capacity, unclear mandates, limited funding	Mentions of counties’ responsibilities, capacity-building, revenue use, promised facilities	General references to “government” without specifying counties
	National level	Roles of national ministries and parastatals (KFS, KMFRI, KMA, Blue Economy Ministry) and policy silos	References to central government duties, duplications between KFS/KMFRI, policy incoherence	Purely county-level complaints
	Interlevel relationship, counties	Interactions between counties and local actors (BMUs, traders), ad hoc arrangements, service provision	Specific accounts of county–BMU negotiations, cess arrangements, grievances	Generic statements on overlaps
	Interlevel relationships, BMU	BMUs’ role coordinating with multiple agencies, confusion post-devolution, conflicts	BMU experiences of multiple levies, MoUs with counties, lack of fora	General mentions of BMUs without institutional context
Data collection	County level	County officers’ role in registering actors, collecting data; issues with IDs, absence of PINs	Mentions of county-level registration processes, survey plans, cess records	BMU- or KFS-led data activities
	BMU level	BMUs’ role in daily catch data collection, submission to counties; resource gaps	References to BMUs collecting/analysing catch data, lack of scales, non-dissemination	General statements on weak data systems
Revenue collection	BMU level	BMU by-laws, levies on landing, registration fees, welfare spending	Specific charges, use of repealed regulations, social auditing	General statements about revenue shortfalls
	County level	County-level fees/cess on fish landed, vessel licensing, aquaculture charges	Mentions of cess per Kg, vessel registration, contribution to county revenue	BMU- or national-level revenue
	National level	National government/KFS levy framework, Fish Levy Trust Fund; donor reliance	Statements on KFS collections, earmarking to CRF, foreign vessel licensing	County/BMU levies
Compliance	*(no sub-theme)*	Patterns of adherence/resistance to licensing, tax obligations, seasonal/gear restrictions	Mentions of cultural resistance, low PIN uptake, targeted vs blanket enforcement, smuggling, political interference	General dissatisfaction without compliance angle
Support for the fisheries sector	*(no sub-theme)*	Incentives, exemptions, training programmes, donor projects; their intended and unintended effects	References to aquaculture incentives, SACCO plans, subsidies, donor facilities	Complaints about governance without support angle

## Data Availability

The data that has been used is confidential.

## Appendix A

..

## Appendix B Codebook

This appendix presents the thematic codebook developed during the analysis of interview and focus group data. The codebook documents the major themes and sub-themes that structured the analysis, as well as their definitions, inclusion and exclusion criteria. It reflects the question-guided thematic synthesis approach described in the methodology: responses to interview questions were collated, read repeatedly, and grouped inductively and deductively into thematic categories informed both by the interview guide and by emerging patterns in the data (Gläser and Laudel, 2013, Gale et al., 2013, Borkan, 2022). The codebook should not be interpreted as a rigid coding dictionary, but rather as a transparent record of the analytic scaffold used to organise and interpret the data. In line with interpretive qualitative traditions, the emphasis was on immersion, reflexivity and narrative integration rather than formal reliability testing.

## Footnotes

1 A general definition of resource rent is the excess economic value arising from the exploitation of a natural resource left after all exploitation costs, including compensation to all factors of production, have been taken into account.

2 Throughout the manuscript, we will frequently refer to taxes, fees, charges and cess. While they all flow to different level of governments, there are important differences between them. While they are all compulsory payments made by individuals or institution to a government body, they differ in scope and in what is obtained in return. Taxes are unrequited, that is no service is rendered by the government directly in return for this payment. Conversely, fees are payment made in return for the delivery of a specific service, while charges are payment impose for services used or access to public infrastructure. A cess is instead a tax levied for a specific purpose, that is one whose revenue is earmarked for a dedicated use. Interested readers can find an in-depth discussion of the role of some of these instruments in the context of environmental fiscal reforms in Cottrell and Falcão (2018).

3 Kenya’s overall tax structure closely resembles that of other anglophone countries worldwide. Taxes levied on the domestic economy includes those on the profit of incorporated entities (11.56% of total revenue collected in the 2024), on the salary of those formally employed (22.57%), on capital gains (0.34%) as well as value-added taxes on domestic consumption (13.05%). These are complemented by trade tariffs and value-added tax on import (32.88%) and from non-tax revenue collected by a variety of ministries, department and agencies providing direct services to the citizenry (7.65%). See KRA (2024) for the most detailed publicly available breakdown of revenue collection.

4 Fish cesses are levied directly on fishers at the moment of landing.

5 ‘“Fisher” means every person employed or engaged in any capacity or carrying out an occupation on board any fishing vessel, including persons working on board who are paid on the basis of a share of the catch but excluding pilots, naval personnel, other persons in the permanent service of a government, shore-based persons carrying out work aboard a fishing vessel and fisheries observers’ (The Fisheries Management and Development Act, Ch. 378: 13).

6 Institutional bricolage is process through which institutional arrangements arising from different contexts – such as formal rules derived from legislation and informal systems based on established social practices – are mixed to fit the existing circumstances regardless of their original purpose.

7 Elite capture describes a situation in which individual with high social status diverts public or group resources towards their private gain rather than towards their destined use. In the fisheries sector, this phenomenon is especially relevant for women stakeholders, who are historically under-represented in the sector, and for whom BMUs are crucial to influence fisheries management and enjoy the results of their labour (see Geheb et al. 2008; Matsue, Daw and Garrett 2014).

8 Recently concluded negotiations at the World Trade Organization ban subsidies to fleets that are explicitly targeting overfished stocks − see Tipping (2020) and Tipping and Irschingler (2021).

9 Namely, we reviewed the Fisheries Act Chapter 378 of 1991 (2012 Revision), the Fisheries (Beach Management Unit) Regulations of 2007 (2012 Revision), the Fisheries Management and Development Act of 2016, the Marine Fisheries (Access and Development) Regulations 2022, the Fisheries Management and Development (Marine Fisheries) Regulations 2024, and the Fisheries Management and Development (Fish Levy Trust Fund) Order 2024.

10 This specific period was chosen as we wanted to focus on the years following the implementation of the Fisheries Management and Development Act of 2016.

11 The choice of conducting only one FGDs was mainly dictated by the fieldworks’ timeline and logistics, which required the author to visit different part of Kenya over the course of ten days. In this context, we could organise one FGDs in Mombasa, were one of the authors was based, but did not manage to secure one in Homa Bay, where we could only interview 3 members of the beach management unit.

12 The resulting structure, including the themes and sub-themes which emerged, are documented in Appendix.B, which also includes general definitions and inclusion/exclusion criteria. This codebook should be intended as a rigid dictionary, but rather as a transparent record of analytic decisions made during the analysis phase of the study.

13 Despite its name, KFS also oversees the aquaculture sector. This has its own section of the Act (Part VIII), as well as separate licensing requirements covered in Part X.

14 It is also interesting that the interpretation of the meaning of ‘fisheries’ we were given by both central institutions (‘*fisheries refers to managing the fish as a product after it is removed from water*’, KFS official), and by county governments (‘*fisheries is what happens when the fish leave the water*’, Homa Bay County official) is similar to that provided by some interviewees to Mudliar and O’Brien (2021), but does not find any support in the Act.

15 Interview with official from KMFRI.

16 Interviews with officials from KFS and KMFRI. While the information on our possess does not allow us to extensively elaborate on the direct impact of the reliance on donor funding on KFS and KMFRI governance and accountability, we cannot exclude that a connection exist. This is especially due to the fact that the availability of donors’ funding clearly has an impact on what activities can be carried out, as exemplified by this quote from a KMFRI official “*We are trying to do research to guide policy, but we have to seek funds from donors, for example to do a catch assessment on Lake Turkana, which is the biggest water body in Kenya. It is a logistical challenge; it requires a lot of funding*.”.

17 Interviews with official from KMFRI, KFS, and the chairman of the BMUs Association.

18 Interviews with official from KFS and the chairman of the BMUs Association.

19 Interview with official from KFS.

20 Interview with official from KFS.

21 “*[F]isheries licensing is not supposed to be for revenue collection, it’s supposed to be a management measure… I license you when there is a space. When the stock is enough for 100 people, I license 100. If this stock is proved to be getting down, we license 50 boats only.”* Interview with official from KFS.

22 Interview with official from Mombasa County.

23 Interview with official from Mombasa County.

24 We cannot however exclude that additional revenue from fisheries was misclassified and reported under other revenue headings.

25 Interview with officials from Homa Bay County and fishers from Alero BMUs.

26 Interviews with officials from KFS and KMFRI.

27 Interviews with officials from Homa Bay County government.

28 ‘*The lack of alternative revenue sources is also a reason – there is not much outside of the primary sector that counties can get money from, so they are taxing these on the road, at the market, when they leave town, and then the fishermen wonder what is left for them*’, interview with official from KMFRI.

29 ‘*The fisheries sector is highly interconnected: if you focus on processors, the cost will trickle down; if you focus on transporters, it will go up and down* [along the value chain]’, interview with fishers from Alero BMU.

30 Interviews with senior representative of BMUs association, fishers at Alero BMU, and official from KFS.

31 Interview with senior representative of BMUs association.

32 Interview with senior representative of BMUs association.

33 Interview with senior representative of BMUs association.

34 Interview with fishers at Alero BMU, and FGD with Mombasa Old Town BMU.

35 Interview with officials from KFS, KMA, and Homa Bay county government.

36 “*Fisheries management is very dependent on BMUs: they are on the ground, they are the ones who do the fishing, they are the one who educate and sensitise to ensure that* [fishing] *is sustainable. So, we depend on the BMU so much, for collection and similar activities*” interview with KFS official.

37 “*No one coordinate the sector either – it was up to the BMUs in Homa Bay to organise a convention about the sector, inviting the KFS, the County Government and the State Department, and it was in this context that we gave county governments the power to collect from the sector in exchange for receiving some services*”, interview with senior representative of the BMUs association.

38 Interviews with officials from KRA.

39 Interviews with officials from KRA.

40 Interviews with officials from KRA, KMA, and Mombasa County government, senior representative of the BMU association, fisher from Alero BMU, and FGD with Mombasa Old Town BMU.

41 Interview with officials from Mombasa County government.

42 ‘[Fisherfolks] *have low adaptation to change, and fishing is a culture: ‘If my grandfather did not need a license to fish, why should I?*’, interview with official from Mombasa County government; ‘*Why would* [fisherfolks] *want one* [PIN], *they would have to file a return every month – most of which would be nil – and be penalised if they don’t*… [KRA] *only come to ask us money for penalties and for non-filing*’, interview with fisher from Alero BMU; ‘[Boat owners] *do not want to have anything to do with the KRA: they have to pay excise duty on nets, there are levies on engines, the import tariff on the timber of the boats has been increased – this is enough for them*’ interview with senior representative of BMU association.

43 Kenyans accruing any amount of income have to possess a PIN.

44 Interviews with officials from Mombasa and Homa Bay County governments, and fishers from Alero BMUs.

45 Interview with official from KMA. However, it must be noted that there are currently no inter-agency information protocols between the KRA and KMA that allow this information to be shared.

46 Only 25.4 per cent and 18.7 per cent of taxpayers managed by the Mombasa and Nairobi Tax Offices respectively ever make a payment. This falls to 4.7 per cent among those managed by smaller tax offices.

47 Interviews with KRA officials.

48 Interviews with KRA officials.

49 Interviews with senior representative of the BMUs association and fishers from Alero BMU.

50 Interview with official from KMFRI.

51 Interviews with officials from KFS, KMFRI, and Mombasa County government, with senior representative of the BMUs association, with fishers from Alero BMU, and from FGD with Mombasa Old Town BMU.

52 Interviews with officials from Mombasa County governments, the chairman of the national BMUs association

53 Interviews with officials from KFS and KMFRI. The increased complexity of carrying out survey frame in a context of decreasing funding was also commented on by a senior representative of BMUs association.

54 ‘*No one comes to the beach to do data management though – we come to fish! I know that the data we provide is the only thing that can inform policy and can help our case. But the government is not investing much in data collection. You count on me to report the data, but you do not buy me a scale, so any secretary can cook the books upwards or downwards depending on their objective’*, interviews with fishers from Alero BMU.

55 ‘*We have to license with everyone, everyone sends their staff to the BMUs, everyone stops us to check us… everyone wants to take money from the fishermen − but no one is investing enough in* [the sector]’, interview with senior representative of BMUs association; ‘*KMA, KFS, and the coast guard are all patrolling the lake; all of these actors are active in the sector, all of these actors are asking of us*’ interviews with fishers from Alero BMUs; FGD with Mombasa Old Town BMU.

56 Interview with official from KMFRI.

57 Interview with senior representative of the BMUs association.

58 Interviews with officials from Homa Bay County and KMFRI, fisher from Alero BMU, senior representative of BMUs association, and FGD with Mombasa Old Town BMU.

59 Interviews with official from KFS, fishers from Alero BMU, and FGD with Mombasa Old Town BMU.

60 ‘*You cannot see our turnover because we are enemy. We do not normally have a long conversation with the KRA, because they will look at my catch and then ask to implement a lifestyle audit.*’ ‘*The mentality of the KRA is the mentality of the old Kenyan Police: have you paid? This is what you have to pay, if you don’t pay, this is the penalty, I’ll chase you! If you don’t pay, I’ll arrest you!*’. ‘*We wanted electricity at one of the BMUs, as well as a bank account: we walked into a cybercafé to get a PIN, and after a while we got millions of shillings in penalties for non-filing – who told us that we had to nil-file? Who told the KRA that we watch TV, or that we read newspapers? Who has ever seen a KRA official?*’ interviews with fishers from Alero BMU; interview with senior representative of the BMU association.

61 Interviews with officials from the KRA.

62 Interviews with officials from KRA, KMFRI, Mombasa County government, fisher from Alero BMU and the chairman of the national BMUs association.

63 Interview with official from the KMA.

64 *‘Politicians look at the features of their voters and protect them by any means. So, if anything is supposed to be changed that affects their voters in terms of harvesting as much as they can from the sea or the lakes, they will object*’ interview with official from KFS.

65 ‘*Counties also have parallel fees for registration of vessels and license for fishing that are normally three times higher, five times or more* [than those charged by KFS]’, interview with KFS official.

66 Interview with KFS official.

67 *‘There has been a push and pull between the counties and the national government, because public participation is key in our constitution. When the counties, the fishers and the politicians at the county level see a step forward in the process of attaining new regulations, they pull us back’* (interview with KFS official).

68 ‘*Fishing is seen as benefiting a certain political* [group]. *Fishing is done mainly in opposition area, so there is a feeling that people on top of the government* [wondered] *why would we give them any economic incentive?*’, interview with official from KMFRI.

69 Interview with official from KMFRI.

70 Interview with official from KMFRI.
